# Effects of a Course-Based Yoga Intervention on Perceived Stress and Mindfulness Among Nursing Students: A Quasi-Experimental Mixed-Methods Study

**DOI:** 10.3390/healthcare14172845

**Published:** 2026-09-04

**Authors:** Tuğba Özdemir

**Affiliations:** Department of Nursing, Faculty of Health Sciences, Istanbul Gedik University, Istanbul 34858, Türkiye; tugbaozdemir321@gmail.com

**Keywords:** nursing students, yoga, mindfulness, perceived stress, psychological well-being, mind–body intervention, mixed methods

## Abstract

**Background and Objectives**: Yoga has been proposed as a supportive intervention to enhance mindfulness and reduce stress among nursing students, but evidence from mixed-method studies remains limited. This study aimed to examine the effects of a 12-week course-based yoga intervention on mindfulness and perceived stress and to explore students’ experiences of practicing yoga. **Materials and Methods**: A total of 42 second-year nursing students at a university in Istanbul, Türkiye, participated in a mixed-methods study, with 22 students enrolled in the elective Health and Yoga course constituting the intervention group and 20 students enrolled in another elective course constituting the control group. Group allocation was non-randomized and determined by students’ elective-course enrollment. The intervention group attended weekly 90 min yoga sessions for 12 weeks, delivered by a certified instructor (E-RYT 200). Quantitative outcomes were measured using the Mindful Attention Awareness Scale (MAAS) and Perceived Stress Scale (PSS), with baseline-adjusted ANCOVA and change score analyses performed to control for pre-test differences and gender. Effect sizes (partial η^2^) were reported, and 95% CIs were calculated for the mean differences in change scores. Qualitative data were collected via semi-structured interviews and analyzed thematically, integrating findings with quantitative results. **Results**: Baseline-adjusted ANCOVA revealed greater improvements in mindfulness (F(1, 39) = 29.686, *p* < 0.001, partial η^2^ = 0.432) and perceived stress (F(1, 39) = 158.293, *p* < 0.001, partial η^2^ = 0.802) in the intervention group, controlling for baseline scores. Change score analyses confirmed these findings: mindfulness increased (MD = 10.68, 95% CI [6.62, 14.75], *p* < 0.001) and perceived stress decreased (MD = −11.40, 95% CI [−13.32, −9.48], *p* < 0.001) significantly more in the intervention group than controls. Qualitative findings complemented the quantitative results, indicating perceived physical (flexibility, posture, pain reduction), mental (positive emotions, awareness, emotion regulation), and social (motivation, interaction) benefits of yoga. Some participants reported hesitation in applying yoga in nursing practice due to cultural barriers. **Conclusions**: The findings suggest that participating in a course-based yoga program may help improve mindfulness and support stress reduction among nursing students. Qualitative findings complemented the quantitative results by providing insight into students’ perceived physical, mental, and social experiences of yoga. Given the small, single-center, and self-selected sample, these findings should be interpreted cautiously. Larger, multicenter randomized studies with longer follow-up are needed to confirm these findings and assess their generalizability.

## 1. Introduction

Nursing undergraduate students face many physical, mental and social challenges throughout their education. These challenges may contribute to mental health problems such as stress, anxiety, and depression, which can negatively affect sleep and academic performance [1,2,3]. Nursing student cohorts often include both traditional-entry and mature students, whose adaptation to the academic and clinical demands of higher education may differ [3]. In a longitudinal study conducted among nursing students in Türkiye, psychological distress, anxiety, and perceived stress increased significantly over the course of nursing education, with an increase in depressive symptoms also observed by the fourth year [4]. The nursing education curriculum includes intensive clinical practice in addition to theoretical courses, requiring students to adapt to both academic and practice-based demands unlike many predominantly classroom-based programmes. Students encounter an increasing theoretical knowledge load and clinical responsibilities as they progress through their education, which raises their stress levels. Stress experienced during clinical practice has been found to negatively affect students’ professional competence, reduce self-confidence, and make it difficult to develop professional skills [5]. Especially during clinical practice periods, students must cope with factors such as patient care, lack of professional knowledge, and fear of making mistakes. These stress factors can negatively affect students’ academic achievement and professional competence [6]. Therefore, the intensity of nursing education and the demands of clinical practice should be considered when developing strategies to support students’ psychological well-being.

Previous studies have highlighted that only a small number of nursing students receive professional support and that many experience untreated mental health problems [7,8,9]. Stress has been identified as the most important factor affecting depressive symptoms in nursing students, with increased self-efficacy and emotion-focused coping shown to have a positive effect on depressive symptoms [10]. Self-efficacy, defined as an individual’s belief in their capacity to execute the behaviors necessary to produce specific performance outcomes, has been widely recognized as a key psychological resource in coping with stress [11]. Nursing students have been found to have high stress levels, with the most significant contributing factors being, in order of importance, financial issues, curriculum intensity, clinical problems, trust issues, and other personal concerns [12]. These findings highlight the need to support nursing students not only academically but also psychosocially. At this point, complementary and integrative health practices, especially mind–body approaches, may offer supportive strategies for managing stress. From a psychological perspective, stress among nursing students can be understood through the transactional model of stress and coping [13], which conceptualizes stress as arising from individuals’ appraisal of environmental demands in relation to their perceived coping resources. Recent longitudinal evidence in student populations continues to support this framework, showing that appraisal and coping processes prospectively predict stress and related mental health outcomes [14]. Coping responses may involve problem-focused strategies aimed at managing the source of stress or emotion-focused strategies aimed at regulating emotional responses to stress [13]. Interventions that strengthen coping resources such as mind–body practices may therefore buffer the psychological impact of academic and clinical stressors. These concerns highlight the potential value of accessible supportive strategies that can be incorporated into nursing education.

Yoga, a complementary and integrative health practice based on the mind–body connection, has historical roots dating back thousands of years and positive effects on an individual’s physical, social, and spiritual well-being, distinguishing itself from other physical activities through its emphasis on the unity of body, mind, and breath. Yoga is also recognized within nursing practice frameworks, such as the Nursing Interventions Classification (NIC), as an independent mind–body intervention, though its psychological mechanisms are of broader relevance beyond any single professional discipline [15]. Mindfulness, defined as the capacity to maintain non-judgmental, present-moment awareness of one’s thoughts, emotions, and bodily sensations [16,17], is a relevant psychological outcome of mind–body practices such as yoga. Recent evidence continues to support the relevance of mindfulness-based approaches specifically within nursing education, linking them to reductions in stress, anxiety, and depression and improvements in emotional regulation among nursing students [18]. Traditional Hatha yoga consists of meditation, breathing exercises, and yoga asanas (yoga poses) [19,20,21,22,23]. In studies conducted with nursing students, yoga has been found to increase self-compassion [24,25] and reduce stress and anxiety levels [26]. Mindfulness-based meditation has been found to reduce stress and anxiety in nursing students [27,28]. Nursing students’ experience of non-pharmacological interventions that support individual recovery and are carried out under expert guidance during their training may contribute to their own mental health [29]. A systematic review also suggested that awareness-based and complementary approaches incorporated into nursing curricula may help address student burnout [30]. In this context, yoga may be a relevant intervention for supporting psychological well-being and stress regulation among nursing students.

In the literature, Erkin and Aykar were the first to integrate a yoga course into the nursing curriculum in Türkiye [25]. Embedding such practices as elective coursework, as in the present study, offers one pragmatic approach to introducing mind–body strategies within an already demanding nursing curriculum, without requiring changes to core, mandatory course content. In Erkin and Aykar’s study outcomes were evaluated using only quantitative measures. Thus, there remains limited evidence combining quantitative assessment of mindfulness and perceived stress with qualitative exploration of students’ experiences of a course-based yoga intervention. First-year students were not included, as their curriculum is predominantly theory-based with limited clinical exposure. Second-year students were specifically selected because, following a first year focused primarily on foundational and theoretical courses, they experience both theoretical and clinical demands as clinical practice intensifies across both semesters, making the second year a critical period in nursing education. Therefore, this study aimed to examine changes in perceived stress and mindfulness among nursing students participating in a 12-week course-based yoga intervention and to explore their experiences of practicing yoga.

### Research Question and Hypotheses

RQ1. Does participation in a 12-week course-based yoga intervention affect perceived stress among nursing students?

RQ2. Does participation in a 12-week course-based yoga intervention affect mindfulness among nursing students?

RQ3. How do nursing students describe their experiences of participating in the course-based yoga intervention, and how do these experiences complement and contextualize the quantitative findings?

**H_1_:**  *Nursing students participating in the yoga intervention will show greater reductions in perceived stress than controls.*

**H_2_:**  *Nursing students participating in the yoga intervention will show greater increases in mindfulness than controls.*

## 2. Materials and Methods

### 2.1. Research Design

This study employed a convergent mixed-methods design, in which quantitative data (pre-test–post-test control-group quasi-experimental component) and qualitative data (qualitative descriptive component) were collected during the same overall study phase, with qualitative interviews conducted immediately following the quantitative post-test using a predetermined interview guide. A convergent design was considered appropriate for this study because it allowed the statistical effects of the intervention to be corroborated and enriched by participants’ own accounts of their experience, providing methodological triangulation between numerical outcomes and lived experience. The quantitative and qualitative strands were analysed separately and then merged and compared at the interpretation stage, consistent with a convergent parallel design [31].

### 2.2. Research Population/Sample

The research population consists of second-year nursing students (*n* = 42) at a foundation university (a non-profit, privately funded higher education institution operating under the supervision of Türkiye’s Council of Higher Education, distinct from both state and for-profit private universities). Students who enrolled in the Health and Yoga course as an elective course during the spring semester of 2025–2026 were included in the yoga group (*n* = 22), and students who enrolled in another elective course were included in the control group (*n* = 20). The control group students were enrolled in a different elective course of equal duration (14 weeks), which began and ended concurrently with the Health and Yoga course but did not include any stress-reduction, mindfulness, or relaxation-based content. This comparison group, held in a classroom format with similar weekly contact hours, was included to provide an active but content-neutral control condition. Group allocation was non-randomized and was determined by students’ elective-course enrollment; students were not assigned to groups by the researchers. A priori power analysis, conducted using an Analysis of Covariance (ANCOVA) framework in G*Power (version [3.1.9.4]; Heinrich Heine University, Düsseldorf, Germany) 2 groups, 1 covariate), indicated that a minimum total sample size of 41 participants would be required to detect a large effect size (f = 0.45) with 80% power at α = 0.05; a total of 42 participants were recruited. In the qualitative dimension of the study, criterion sampling was used as the sampling method. The criterion was students who took the ‘Health and Yoga’ course and were in the yoga group. All 22 students in the intervention group were invited to participate in the qualitative component, and all voluntarily participated. Thus, no refusals or withdrawals occurred during qualitative data collection. As all eligible participants (*n* = 22) were included, sample adequacy was determined by total population inclusion rather than data saturation; consequently, saturation criteria were not applicable to this study.

Inclusion criteria for the study: No chronic/neuro-muscular/respiratory system-related illnesses that prevent yoga practice, proficiency in Turkish, and willingness to participate in the study.

### 2.3. Data Collection Tools

The study data were collected using a Personal Information Form, Perceived Stress Scale, Mindful Attention Awareness Scale, and a semi-structured form.

Personal Information Form: The form created by researchers in line with the literature includes questions for students regarding their gender, physical activity, and perceived health status [24,25].

Perceived Stress Scale: A scale developed by Cohen et al. to measure how stressful individuals perceive events in their lives [32]. The scale consists of 14 items in a 5-point Likert type. The items on the scale are scored between ‘Never (0)’ and ‘Very often (4)’. Items containing positive statements (4, 5, 6, 7, 9, 10, 13) are scored by reversing the scale. The total score on the scale ranges from 0 to 56. A score of 0–26 indicates low stress, 27–41 indicates moderate stress, and 42–56 indicates high stress. The internal consistency coefficient of the scale is 0.84, and the test–retest reliability coefficient is 0.87 [33]. In this study, the internal consistency coefficient was calculated as 0.76 (pre-test) and 0.78 (post-test).

Mindful Attention Awareness Scale: The Turkish adaptation of the scale developed by Brown and Ryan [17] was carried out by Özyeşil et al. The scale is a 15 items, 6-point Likert that measure the general tendency to be aware of momentary experiences in daily life and to pay attention to these experiences. The item factor loadings for each item of the scale range from 0.48 to 0.81, and the increase in scores shows an increase in the level of conscious awareness. The minimum score that can be obtained from the scale is 15, and the maximum score is 90. The scale’s internal consistency coefficient is 0.80, and its test–retest reliability is 0.86 [34]. In this study, the internal consistency coefficient was calculated as 0.75 (pre-test) and 0.83 (post-test).

### 2.4. Intervention

After group allocation based on elective-course enrollment, baseline quantitative data were collected from both groups. Students in the yoga group were provided with theoretical information on the history and philosophy of yoga, the effects of yoga on health, evidence-based research examples applied within the scope of nursing initiatives related to yoga, the alignment of basic yoga poses and their effects on the body, pranayama and its effects on health, and meditation and its types, within the scope of the ‘Health and Yoga’ content during the first two weeks. The yoga intervention was embedded within a 14-week elective Health and Yoga course. The first two weeks focused on theoretical content, followed by 12 weeks of yoga practice (Weeks 3–14). The yoga intervention was conducted once per week for approximately 90 min over 12 weeks. The yoga intervention was conducted by a researcher who is a public health nurse and also holds an E-RYT 200 certification (Basic Vinyasa Yoga Instructor Certification). Attendance was monitored through course attendance records. All 22 participants completed the 12-week yoga intervention, and no loss to follow-up occurred. No adverse events were reported in the intervention. Some participants reported difficulty in entering or maintaining certain poses, which reflects individual variability in physical adaptation. For those who had difficulty entering or maintaining poses, the instructor adjusted the difficulty level of the poses to make it easier to enter/maintain them. The instructor participated in the classes live. No structured home practice was assigned. Students practised basic Hatha Yoga poses as part of Vinyasa Yoga. All poses were demonstrated at a basic beginner level, and the practice began with equal-length breaths.

Guided meditation was followed by warm-up movements involving movement of the spine in all directions before moving on to the asanas. The flow of asanas was followed by cooling poses, and finally, the practice was completed with a relaxation pose (savasana). The content of the study and the yoga sequence are presented in Appendix A. Intervention fidelity was supported by delivery of all yoga sessions by the same certified instructor and adherence to the standardized session sequence described in Appendix A. At the end of the yoga intervention, the final test quantitative data were collected face-to-face from both groups. After the quantitative data were collected, the researcher used a semi-structured interview form to gather students’ opinions about the programme in the yoga class, and the data collection process was completed. During the individual interviews with students in the yoga class, which lasted approximately 15 min, no one other than the researcher and the student was present in the classroom. The data were recorded on paper according to the students’ opinions. The researcher who conducted the interviews was also the instructor who delivered the yoga intervention. The control group continued to attend their alternative elective course and did not receive any yoga-related intervention.

### 2.5. Data Analyses

The quantitative data of the research were transferred to the SPSS Statistics (version 26.0; IBM Corp., Armonk, NY, USA) statistical package programme in a computer environment and analysed. Mean, standard deviation, median, number, and percentage were used in the evaluation of descriptive data. The Kolmogorov–Smirnov test was used to examine the suitability of the data for normal distribution. Baseline-adjusted ANCOVA was prespecified as the primary quantitative analysis to examine between-group differences in post-intervention MAAS and PSS scores while adjusting for the corresponding pre-intervention scores. Gender was included as a covariate. Change scores were calculated as a sensitivity analysis because they do not rely on the assumption of homogeneity of regression slopes required by ANCOVA; the convergence of results across both approaches supports the robustness of the observed intervention effects. As a supportive analysis, change scores were calculated by subtracting pre-intervention scores from post-intervention scores, and between-group differences in change scores were examined using independent-samples *t*-tests. 95% confidence intervals (CIs) were calculated for the between-group mean differences in change scores. Effect sizes for change-score comparisons were calculated as r from the independent-samples *t*-test results, with 95% CIs estimated using Fisher’s z transformation. Non-parametric tests were additionally conducted as supportive analyses. Prior to conducting ANCOVA, the assumptions of normality of residuals (Shapiro–Wilk test), homogeneity of variance (Levene’s test), linearity, and homogeneity of regression slopes (covariate × group interaction) were examined. The level of significance was accepted as 0.05. Given the exploratory nature of the study and the limited number of outcomes, no formal correction for multiple comparisons was applied. Effect sizes were reported using partial eta squared (η^2^) for ANCOVA models.

Qualitative data were collected after the quantitative post-test within the qualitative descriptive component of the convergent mixed-methods design and analysed using thematic analysis. The researcher conducted individual face-to-face interviews (a PhD-level nurse educator who also delivered the yoga course), each lasting approximately 15 min and following a brief semi-structured interview guide with predetermined questions focused on participants’ experiences of the yoga intervention, with follow-up probes used as needed (e.g., asking participants to elaborate on specific experiences they mentioned). Responses were documented in written form directly during the interview rather than audio-recorded and transcribed verbatim. All 22 participants from the intervention group were interviewed, reflecting total population sampling rather than a saturation-based approach. No repeat interviews were conducted. Thematic analysis was guided by Braun and Clarke’s six-phase reflexive thematic analysis framework, which was selected as it is well suited to identifying patterned meaning within a qualitative descriptive design without requiring a specific theoretical or interpretive stance, consistent with the descriptive (rather than interpretive-phenomenological) aim of this component [35]. The data obtained through individual interviews were documented in a computer environment. Themes were derived inductively from the data and guided by the research questions. Data collection and analysis were conducted iteratively to refine emerging themes. Coding was performed based on the participants’ responses. The analysis of the qualitative data of the research was carried out manually by the researcher without using any computer-assisted qualitative data analysis programme. After each interview, participants’ written responses were read back to them, and they were asked whether they wished to confirm, revise, or add to their statements before finalizing the data (member checking); no participant requested changes. Participants’ personal information has been kept confidential, and data has been identified using only code numbers. The coding process was conducted by the researcher, but themes were reviewed multiple times to enhance consistency. To enhance trustworthiness, findings were supported by direct participant quotations and interpreted alongside quantitative results (methodological triangulation). Because the researcher also delivered the intervention and conducted the qualitative data collection and analysis, potential researcher influence and social desirability bias were acknowledged. Before the course and during the research participation process, students were informed that the study was a scientific investigation and that openly sharing their personal experiences would contribute to a more accurate understanding of the intervention. They were also informed that the course emphasized personal experience and individual well-being alongside academic learning, and that their research responses would not affect their course evaluation or academic standing. Participation in the qualitative component was voluntary, and individual interviews were conducted in a private setting. Reflexive notes were maintained during the analysis to critically examine the researcher’s preconceptions and potential influence on interpretation. Coding decisions were colour-coded within the analysis document, with a brief written rationale recorded for each decision, while a separate research journal documented instances where initial coding decisions were reconsidered, merged, or reassigned to different themes during the iterative analytic process. For example, physical benefit statements initially coded separately under sub-themes for flexibility and pain were later reconsidered and consolidated within a broader “physical experience” sub-theme during iterative review. This provided a transparent audit trail of the evolving thematic structure. As no co-analysts were involved, this reflexive record served as the primary mechanism for enhancing the trustworthiness and rigour of the thematic analysis. As a Turkish nurse educator aware of the potential cultural and religious sensitivities associated with yoga in the local context, the researcher deliberately framed and delivered the intervention around its secular, evidence-based health and wellness dimensions rather than its spiritual origins. The Qualitative Research Reporting Consolidated Criteria (COREQ) guidelines have been used as a guide for reporting qualitative data [36]. It follows the Template for Intervention Description and Replication (TIDieR) guidelines for reporting yoga research [37]. Given the quasi-experimental design, results should be interpreted with caution in terms of causal inference.

### 2.6. Ethics Approval and Consent to Participate

Non-Interventional research ethics approval was obtained from the Non-Interventional Research Ethics Committee of Istanbul Gedik University (Date: 7 April 2025, Decision No: 2025/4). Written informed consent to participate was obtained from all nursing students prior to their enrolment in the study. Written permission to use the scales employed in the study was obtained via email from the original developers/copyright holders. The study was conducted in accordance with the principles of the Declaration of Helsinki.

### 2.7. Use of Generative AI

During manuscript preparation, the author used Claude (Anthropic) in a supportive capacity to suggest approximately two contextual sentences added to the Section 1 and Section 4. The author reviewed, edited, and incorporated these suggestions at her discretion. The study design, data collection, statistical and qualitative analyses, interpretation of findings, and all scientific content of this manuscript are entirely the author’s own work.

## 3. Results

The majority of participants were female in both the intervention (59.1%) and control groups (80%). Physical activity levels were mostly moderate, and most students reported good health status. 63.6% of the students described their health status as good. In the control group, 80% of the students were female, 30% had physical activity levels that were neither sufficient nor insufficient, and 50% reported good health. Groups did not differ significantly at baseline in terms of gender (*p* = 0.190), physical activity level (*p* = 0.483), or perceived health status (*p* = 0.167) (Fisher’s exact test for gender and physical activity; χ^2^ test for perceived health; Table 1).

Baseline-adjusted ANCOVA results showed a statistically significant effect of the intervention on mindfulness, with an adjusted mean difference of 10.79 points (95% CI [6.78, 14.79]) favoring the intervention group (F(1, 39) = 29.686, *p* < 0.001, partial η^2^ = 0.432), controlling for baseline scores. Similarly, a significant effect was found for perceived stress, with an adjusted mean difference of −11.69 points (95% CI [−13.56, −9.81]) (F(1, 39) = 158.293, *p* < 0.001, partial η^2^ = 0.802), indicating a substantial reduction in stress levels in the intervention group compared to the control group. Additional analyses controlling for gender indicated that gender was not a significant covariate for mindfulness (F(1, 38) = 2.972, *p* = 0.093, partial η^2^ = 0.073) or perceived stress (F(1, 38) = 1.573, *p* = 0.217, partial η^2^ = 0.040) (Table 2). Residuals were normally distributed for both outcomes (mindfulness: W = 0.964, *p* = 0.205; perceived stress: W = 0.972, *p* = 0.397). However, the assumption of homogeneity of regression slopes was not met for either mindfulness (*p* = 0.001) or perceived stress (*p* = 0.003), and Levene’s test indicated unequal variances for both outcomes (*p* < 0.001 and *p* = 0.044, respectively).

As a robustness analysis, change score comparisons were conducted to examine between-group differences. The increase in mindfulness was significantly greater in the intervention group compared to controls (MD = 10.68, 95% CI [6.62, 14.75], *p* < 0.001). Similarly, perceived stress decreased significantly more in the intervention group (MD = −11.40, 95% CI [−13.32, −9.48], *p* < 0.001). The corresponding effect sizes were large for both MAAS (r = 0.64, 95% CI [0.42, 0.79]) and PSS (r = 0.88, 95% CI [0.79, 0.94]) (Table 2).

Within the intervention group, mindfulness scores significantly increased from pre-test (47.40 ± 12.89) to post-test (57.04 ± 14.36) (*p* < 0.05), and perceived stress scores significantly decreased from pre-test (38.04 ± 4.19) to post-test (26.09 ± 7.25) (*p* < 0.05). No significant within-group changes were observed in the control group for either mindfulness or perceived stress (*p* > 0.05) (Table 3).

As a result of the qualitative data analysis, five themes were identified: motivation for engaging with yoga, embodied and psychological benefits of practice, social dimensions of the practice, translating personal experience into future clinical practice, and challenges and limits of the practice. A joint display was created to integrate qualitative and quantitative findings. The qualitative themes were aligned with quantitative results, demonstrating convergence between increased mindfulness, reduced stress, and participants’ reported experiences (Table 4).

Theme 1. Motivation for engaging with yoga

Students have selected Health and Yoga as an elective course. When asked why they chose yoga, students stated that they were curious about yoga, experience, and believed it would benefit them physically and mentally.

Sub-Theme 1. Curiosity

‘I chose it to find out if it really has the effects that are claimed, because I was curious.’ (Ö20).

‘Because I was curious…’ (Ö10)

‘How it will benefit patients and how we can apply it…’ (Ö12)

Sub-Theme 2. Seeking a new experience

‘A change in routine.’ (Ö7)

‘To have a different experience…’ (Ö8)

‘I wanted to try it.’ (Ö9)

Sub-Theme 3. Anticipated physical/mental benefits

‘To learn about yoga and improve both my physical and mental well-being through the exercises we do…’ (Ö13)

‘I chose this class to achieve physical and psychological relaxation.’ (Ö16)

‘To relax my mind and body.’ (Ö2)

‘…I chose it for my physical and mental health.’ (Ö21)

Theme 2. Embodied and psychological benefits of practice

Students described yoga’s benefits as spanning both bodily and emotional experience, often in the same breath-physical changes such as flexibility and reduced pain were frequently described alongside feelings of calm, awareness, and emotional regulation, suggesting that participants experienced these dimensions as interconnected rather than separate.

Sub-Theme 1. Physical experience (flexibility, vigor, posture, pain)

‘I gained flexibility and posture.’ (Ö1)

‘My mind relaxed and I gained flexibility.’ (Ö2)

‘I felt increased flexibility in my body.’ (Ö9)

‘I felt more vigorous.’ (Ö5)

‘I used to find planks very difficult, but now I can hold the plank position for longer, my inner thigh muscles were tight but now they are more flexible and my feet are flat on the floor.’ (Ö16)

‘I felt more vigorous and healthy.’ (Ö22)

‘It helped me stand straighter.’ (Ö6)

‘Better posture and better mental…’ (Ö13)

‘I saw changes in my posture.’ (Ö21)

‘My muscle pain has decreased’ (Ö10)

‘It helped my body pain’ (Ö14)

‘Especially relaxation and relief in the back area’ (Ö19)

Not all physical experiences were uniformly positive: one participant described an ambivalent experience, reporting reduced shoulder pain alongside a perceived decrease in physical strength and weight loss-‘I lost weight and became physically weaker, and my shoulder pain decreased’ (Ö12)-illustrating that participants’ embodied experiences were not universally positive.

Sub-Theme 2. Psychological/emotional experience (calm, awareness, emotion management)

‘I found peace.’ (Ö1)

‘I am calmer.’ (Ö3)

‘After yoga, I felt mentally relaxed.’ (Ö12)

‘I became a more positive person.’ (Ö13)

‘I feel more mentally refreshed and relaxed.’ (Ö16)

‘Stay calm, stay healthy.’ (Ö18)

‘My anxiety and stress decreased.’ (Ö2)

‘I became more aware.’ (Ö5)

‘It was wonderful to do something for myself and be in the moment.’ (Ö7)

‘I was able to manage my emotions better.’ (Ö10)

‘I thought less.’ (Ö15)

‘I felt calmer and ready to enjoy the day.’ (Ö19)

‘I started thinking more logically.’ (Ö20)

‘I felt like I understood myself better.’ (Ö22)

‘The most beneficial part was the meditation where we listened to ourselves and relaxed our bodies.’ (Ö22)

Theme 3. Social dimensions of the practice

Students described increased self-awareness in social contexts, richer social interaction and motivation, though some reported no perceived social effect.

Sub-Theme 1. Self-awareness in social contexts

‘I feel less ashamed.’ (Ö1)

‘I have gained understanding.’ (Ö3)

‘I was able to speak more comfortably.’ (Ö10)

‘Improved communication.’ (Ö17)

‘I get along better with those around me.’ (Ö21)

Sub-Theme 2. Social interaction and motivation

‘I socialised more.’ (Ö5)

‘My friend and I decided to do yoga and fitness training.’ (Ö9)

‘We moved in harmony because we did it together with friends.’ (Ö16)

‘I had more opportunities to meet and talk with friends I don’t usually talk to much.’ (Ö19)

‘Doing yoga together was both enjoyable and more motivating to exercise.’ (Ö22)

Sub-Theme 3. No perceived social impact

‘It didn’t seem to have any effect.’ (Ö2)

‘It was already good, so I don’t think it had much effect.’ (Ö14)

‘There was no change.’ (Ö20)

Theme 4. Translating personal experience into future clinical practice

Students expressed a wish to incorporate yoga into future patient care based on their personal benefit, but also voiced doubts about its feasibility in clinical settings.

Sub-Theme 1. Perceived relevance to patient care

‘Yes, because it relaxes the mind and body.’ (Ö2)

‘Yes, because it is a beneficial type of exercise and has been proven to work.’ (Ö5)

‘Yes, I think so, because it has a positive effect on things like stress, pain and anxiety.’ (Ö9)

‘Yes, it can be included, because yoga helps reduce pain and alleviate anxiety.’ (Ö10)

‘Yes, I can include it, as it helps them relax.’ (Ö12)

‘Yes, I think so, because I believe it has an effect that can help patients overcome anxiety, fear, and worry.’ (Ö16)

Sub-Theme 2. Perceived barriers to incorporation

‘No, it is very difficult to get our people to do yoga.’ (Ö3)

‘I would love to, but I don’t think it would be feasible.’ (Ö7)

‘No, I don’t think so, because I think patients are very prejudiced and I don’t think they would do it.’ (Ö19)

‘I think it would definitely be very beneficial if included, but I think it would be difficult under the conditions in our country.’ (Ö22)

Theme 5. Challenges and limits of the practice

Students described difficulty entering and maintaining certain yoga poses, though others reported no difficulty.

Sub-Theme 1. Difficulty with poses

‘Balance.’ (Ö1)

‘I had some difficulty with the movements.’ (Ö2)

‘Yes, I had difficulty with the movements because I had never done them before.’ (Ö8)

‘I had difficulty with some of the movements.’ (Ö12)

‘Yes, I did, especially that my arms are very weak and that I need to exercise more often.’ (Ö22)

Sub-Theme 2. No difficulty

‘No, it didn’t, it helped me a lot’. (Ö15)

‘There wasn’t any point where I struggled, everything was just as I wanted it to be.’ (Ö16)

‘No, it didn’t.’ (Ö18)

## 4. Discussion

This study examined nursing students’ mindfulness, perceived stress levels, and yoga experiences before and after yoga practice. Consistent with previous studies in the literature, improvements in mindfulness and perceived stress were observed. The primary aim of this study, however, was to complement these quantitative findings with students’ own accounts of their experience. By integrating quantitative outcomes with qualitative insights, the study offers a more comprehensive understanding of how yoga was experienced by nursing students within an educational context. Given the quasi-experimental, non-randomized design, these findings should be interpreted as associative rather than confirmatory evidence of efficacy.

### 4.1. Quantitative Findings

The mindfulness levels among participating students were moderate at baseline but increased significantly following yoga practice. Similarly, perceived stress levels were moderate before the yoga programme, and were lower afterward. Comparisons with the control group revealed significant improvements in both mindfulness and perceived stress levels. Although there was a gender imbalance between groups, the non-significant gender covariate suggests that gender alone is unlikely to fully account for the observed differences; however, with a sample of 42 participants, the absence of statistical significance does not rule out other unmeasured confounding factors. This finding is consistent with research on other mindfulness-based academic interventions reporting comparable stress reductions across genders [38], although differential gender effects have also been reported elsewhere [39], suggesting that gender effects on intervention outcomes are not uniformly observed across contexts. The consistency of results across ANCOVA and change-score analyses provides some support for the stability of these findings.

Similar studies in the literature have reported positive effects of yoga practice on mindfulness and psychological well-being. After yoga was practised by nursing students at the university, it was found that the students’ levels of mindfulness and self-compassion increased [25]. The effect of yoga on psychological functioning in nursing students has also been investigated, with increases in mindfulness, resilience and life satisfaction, and improvements in perceived stress levels reported following yoga practice [40]. A systematic review examining the effects of yoga on mental health found that yoga fundamentally reduces stress, anxiety, burnout, and depression, demonstrating its potential as a low-cost, accessible intervention for mental health management. At the same time, it was emphasised that future research needs to be improved in order to reveal the long-term effects of yoga [41]. The magnitude of the effects observed in the present study, however, was notably larger than in much of this prior work; this may partly reflect the self-selected nature of participation. Students who chose the elective course may have entered with higher expectancy or motivation for benefit, rather than a uniquely potent effect of the intervention itself. Similar improvements have been reported among other student populations, including pharmacy students [42], general university students [43], and medical students [44], following yoga- or meditation-based interventions. However, these populations differ from nursing students in curricular structure, clinical exposure, and the specific stressors they face, and such findings should not be assumed to generalize directly to the nursing education context without further comparative research.

### 4.2. Qualitative Findings

The reasons students chose the Health and Yoga course were identified as curiosity, desire to gain experience, and anticipated physical and mental health benefits. These motivations are consistent with those reported in other student populations. In a related study, a consciousness-based elective course offered to medical students, in which in-depth interviews were conducted with students, found that motivation for participation centered on learning a complementary and integrative health practice to enhance educational and clinical skills, improve personal health, and seek meaning [45]. Similarly, a study examining Indian university students’ reasons for enrolling in yoga classes found that students were motivated by beliefs that yoga knowledge could benefit others, contribute to personal growth, and align with their academic or research interests [46].

Consistent with these motivations, participants in the present study reported that the yoga programme improved physical flexibility, energy levels, posture, and pain management. In terms of mental and emotional well-being, students reported increased positive emotions, improved self-esteem, greater awareness, and better emotional management. In the social dimension, some students reported increased social interaction and motivation, although others reported no perceived social effect. A comparable mixed-methods study among female university students similarly reported improvements in emotion regulation, self-awareness, anxiety, depression, and perceived stress, as well as physiological changes including increased parasympathetic activity [47]. The broader literature has interpreted such findings as evidence that yoga’s benefits may operate through an integrated mind–body pathway rather than through symptom-specific mechanisms alone; however, because the present study did not assess physiological mechanisms, this interpretation remains a hypothesis rather than a finding demonstrated by the current data.

In the study, students believe that yoga could be included in nursing care plans in the future, given its perceived physical and mental benefits for patients. Some students expressed hesitation about applying yoga in their future professional roles, citing perceived patient prejudice and feasibility concerns within the Turkish healthcare context (e.g., *‘No, it is very difficult to get our people to do yoga,’ Ö3; ‘I think patients are very prejudiced,’ Ö19*). Although participants did not explicitly frame these concerns in terms of cultural attitudes or health beliefs, this hesitation can be interpreted as potentially reflecting broader patterns described in the literature, where individuals are more likely to adopt interventions congruent with their existing cultural schema of health and care [25,48,49].

This interpretation should be regarded as a plausible contextualization of participants’ comments rather than a direct finding, as the interview guide did not specifically probe cultural or religious attitudes. Recent Turkish research has similarly explored culturally adapted, psychospiritual approaches to yoga-based interventions; a mixed-methods study combining yin yoga with teachings from the Bhagavad Gītā reported improvements in anxiety, resilience, and meaning in life among Turkish adults, highlighting the relevance of culturally resonant framing for yoga-based interventions in this context [50]. Framing the present intervention around its secular, evidence-based health and wellness dimensions, rather than its spiritual origins, may similarly help mitigate concerns about compatibility with local religious norms; however, as this was not directly assessed in the present study, it remains a direction for future research. Practical barriers such as limited time, space, and institutional support [51,52] further compound this hesitation. It should be noted that students’ expressed willingness to incorporate yoga into future patient care reflects personal openness and perceived benefit, rather than established clinical competence, defined scope of practice, or evidence regarding the appropriateness of yoga for individual patients; translating personal experience into safe clinical application would require formal training and clinical judgment beyond the scope of this elective course.

The integration of qualitative and quantitative findings strengthens the interpretation of results by linking statistical improvements with participants’ lived experiences, particularly in terms of increased awareness, emotional regulation, and perceived stress relief [31]. This mixed-methods approach represents a key contribution of the present study; rather than simply confirming that yoga is associated with reduced stress and increased mindfulness, it illuminates how students experienced these changes and reveals a tension between students’ personal benefit and their hesitation to translate this into future clinical practice, a dimension that quantitative outcomes alone would not have captured.

### 4.3. Limitations

Several limitations should be considered when interpreting the findings. First, the study employed a non-randomized, quasi-experimental design, in which group allocation was based on students’ elective-course enrollment rather than randomization. This may have introduced self-selection bias, as students who chose the yoga course could have differed systematically in motivation, preference, or prior yoga experience from those who chose the alternative course. Although statistical adjustments (ANCOVA and change score analyses) were applied, and baseline sociodemographic characteristics did not differ significantly between groups, residual confounding related to unmeasured factors such as motivation cannot be ruled out. The observed gender imbalance between groups was not a designed feature of the study but a consequence of the non-randomized, elective-course-based allocation; as gender was not a criterion for course selection, no attempt was made to balance the ratio between groups. This imbalance may be relevant given that mindfulness-based interventions have been reported to show differential effects by gender in some student populations [39]. Although the present analysis found gender to be a non-significant covariate, the imbalance between groups nonetheless represents a further potential source of confounding not fully addressed by statistical adjustment alone.

Second, the relatively small sample size (*n* = 42) and single-center design limit the generalizability of the findings. Small sample sizes are common across yoga intervention research involving nursing students (e.g., Erkin & Şenuzun Aykar, 2021 [25], *n* = 47, Kinchen et al., 2020 [24], *n* = 15); the sample size in the present study was constrained by the enrollment capacity of the elective course rather than recruitment refusal. Future studies could further strengthen methodological rigor by incorporating a three-point (or repeated) measurement design to capture the trajectory of change over time. Cultural factors specific to the study context may also influence the applicability of yoga interventions in different populations. Age was not collected as a study variable; consequently, the potential presence of mature students within the sample, and how their transition experiences may have differed from those of traditional-age entrants, could not be examined.

Third, the intervention was delivered by the researcher, who also served as course instructor and was involved in qualitative data collection and analysis. This dual role created a dependent relationship with participants that may have introduced investigator and social desirability bias, particularly in written responses, given the ongoing instructor-student relationship. Reflexive notes and member checking were used to partially mitigate this risk, but it cannot be entirely excluded.

Fourth, the ANCOVA models did not meet the assumption of homogeneity of regression slopes, indicating that the relationship between pre- and post-intervention scores differed somewhat between groups; Levene’s test also indicated unequal variances between groups. However, the convergence of ANCOVA results with independent change-score analyses, which do not rely on these assumptions, supports the robustness of the observed effects.

Fifth, the intervention was embedded within an elective course incorporating theoretical instruction, yoga practice, pranayama, meditation, and ongoing instructor interaction; therefore, the specific effects of yoga practice could not be isolated from these other course components.

As both outcome measures relied on self-report, responses may have been influenced by social desirability, particularly given the researcher’s dual role as course instructor. Expectancy effects related to self-selected participation cannot be excluded, and outcomes were assessed only in the short term. Intervention fidelity (i.e., consistency of session delivery across the 12-week programme) was not formally assessed, and as both groups were drawn from the same cohort and academic term, contamination between groups cannot be ruled out. Finally, brief written qualitative responses, rather than audio-recorded interviews, may have limited interpretive depth. Statistical adjustments (ANCOVA and change-score analyses) address baseline differences in outcomes but do not resolve these broader design limitations.

## 5. Conclusions

The qualitative findings provided contextual depth to the quantitative results by illustrating how participants experienced changes in awareness, emotional regulation, and physical well-being. The study offers a contextually grounded, experience-informed perspective that complements the quantitative findings, though these experiences represent participants’ self-reported perceptions rather than independently verified outcomes. Students reported perceived physical, mental, and social benefits following yoga practice, alongside difficulties in entering and maintaining certain poses. Some students expressed willingness to incorporate yoga into future patient care, while others cited community perceptions of yoga as a barrier to doing so; as discussed above, this willingness reflects personal openness rather than established clinical competence or evidence of appropriateness for individual patients.

Taken together, the quantitative outcomes, participant-reported experiences, and broader complementary health literature suggest that curriculum-based yoga interventions may hold promise as a non-pharmacological approach to supporting nursing students’ psychological well-being. However, given the small, non-randomized, single-center, self-selected nature of this study, these findings should be considered preliminary and do not by themselves establish sufficient evidence to recommend routine curricular implementation. Larger, multicenter randomized controlled trials, with longer follow-up, assessment of intervention adherence and fidelity, and evaluation of sustained outcomes over time, are warranted before broader conclusions can be drawn.

## Figures and Tables

**Table 1:** Distribution of Students’ Socio-Demographic Characteristics (*n* = 42).

	Yoga Group (*n* = 22)		Control Group (*n* = 20)		Test/*p*
	n	%	n	%	
**Gender**					Fisher’s exact 0.190
Female	13	59.1	16	80	
Male	9	40.9	4	20	
**Level of physical activity**					Fisher’s exact 0.483
Not enough	2	9.1	5	25	
Neither enough nor insufficient	6	27.3	6	30	
Enough	6	27.3	5	25	
Definitely enough	8	36.4	4	20	
**Level of health**					Chi-square 0.167
Uncertain	1	4.5	5	25	
Good	14	63.6	10	50	
Very good	7	31.8	5	25	

**Table 2:** Effects of the Yoga Intervention on Mindfulness and Perceived Stress.

**Baseline-Adjusted ANCOVA Results**					
	**Source**	**F**	***p***	**Partial η^2^**	**Adjusted MD (95% CI)**
**Mindful Attention Awareness Scale**	Group (Yoga vs. Control)	29.686	<0.001	0.432	10.79 (6.78, 14.79)
	Gender	2.972	0.093	0.073	-
**Perceived Stress Scale**	Group (Yoga vs. Control)	158.293	<0.001	0.802	−11.69 (−13.56, −9.81)
	Gender	1.573	0.217	0.040	-
**Change Score Analysis (Robustness Analysis)**					
	**Analysis**	**Mean Difference**	**95% CI**	***p***	
**MAAS**	Change score	10.68	[6.62, 14.75]	<0.001	
**PSS**	Change score	−11.40	[−13.32, −9.48]	<0.001	

Bold formatting is used to distinguish row-group headers (scale names, analysis type) and sub-table column headers from numeric values.

**Table 3:** Distribution of Scale Scores by Group and Time.

		Pre Test (X ± SS)	Post Test (X ± SS)	Z * p
**Mindful Attention Awareness Scale**	**Yoga Group (*n* = 22)**	47.40 ± 12.89	57.04 ± 14.36	**−3.756** **0.000**
	**Control Group (*n* = 20)**	46.80 ± 6.06	45.75 ± 3.29	−0.634 0.526
	**Z †** ***p***	−0.177 0.860	**−2.763** **0.006**	
**Perceived Stress Scale**	**Yoga Group (*n* = 22)**	38.04 ± 4.19	26.09 ± 7.25	**−4.112** **0.000**
	**Control Group (*n* = 20)**	36.75 ± 4.30	36.20 ± 4.00	−1.877 0.060
	**Z †** ***p***	−0.860 0.390	**−4.503** **0.000**	

* Wilcoxon Test, † Mann–Whitney U. Bold formatting is used to distinguish row-group headers (scale names, group labels) from numeric values, and to highlight statistically significant results (*p* < 0.05).

**Table 4:** Integration of Qualitative Themes with Quantitative Findings (Joint Display).

Themes	Sub-Themes	Students	Type of Integration	Quantitative Interpretation
1. Motivation for engaging with yoga	Curiosity Seeking a new experience Anticipated physical/mental benefits	Ö5, Ö7, Ö8, Ö9, Ö10, Ö11, Ö12, Ö13, Ö15, Ö16, Ö17, Ö19, Ö20, Ö21, Ö22	Contextual (not directly linked)	Reflects participants’ initial motivations for course selection. As group allocation was based on elective enrollment rather than randomization, these motivations may also relate to pre-existing interest and cannot be fully disentangled from the intervention’s effects on subsequent MAAS↑ and PSS↓ changes.
2. Embodied and psychological benefits of practice	Physical experience (flexibility, vigor, posture, pain) Psychological/emotional experience (calm, awareness, emotion management)	Ö1, Ö2, Ö3, Ö5, Ö6, Ö7, Ö9, Ö10, Ö12, Ö13, Ö14, Ö15, Ö16, Ö17, Ö18, Ö19, Ö20, Ö21, Ö22	Convergence (psychological sub-theme)/Plausible interpretation (physical sub-theme)	The psychological/emotional sub-theme (calm, awareness, emotion regulation) directly converges with the significant increase in mindfulness (MAAS↑) and decrease in perceived stress (PSS↓) (Table 2), as these constructs closely align with what MAAS and PSS measure. The physical sub-theme (flexibility, posture, pain) was not directly assessed by MAAS or PSS; its association with the quantitative outcomes is a plausible interpretation rather than a directly measured link.
3. Social dimensions of the practice	Self-awareness in social contexts Social interaction and motivation No perceived social impact	Ö1, Ö2, Ö3, Ö5, Ö8, Ö9, Ö10, Ö11, Ö12, Ö14, Ö15, Ö16, Ö17, Ö19, Ö20, Ö21, Ö22	Plausible interpretation	Social experiences were not directly measured by MAAS or PSS. Their potential relationship to improved psychological well-being is offered as a plausible, complementary interpretation rather than direct quantitative confirmation.
4. Translating personal experience into future clinical practice	Perceived relevance to patient care Perceived barriers to incorporation	Ö2, Ö3, Ö5, Ö7, Ö9, Ö10, Ö12, Ö16, Ö19, Ö22	Divergence/tension	Highlights a tension between participants’ personal benefit (aligned with MAAS↑, PSS↓) and their hesitation to translate this into professional nursing practice, pointing to cultural and institutional barriers rather than a limitation of the intervention’s individual-level effects.
5. Challenges and limits of the practice	Difficulty with poses No difficulty	Ö1, Ö2, Ö8, Ö12, Ö15, Ö16, Ö18, Ö22	Divergence/tension	Illustrates that participant-reported physical difficulty coexisted with overall improvements in MAAS and PSS, indicating that positive outcomes were not experienced uniformly or without effort.

MAAS↑ indicates increased mindfulness; PSS↓ indicates decreased perceived stress.

## Data Availability

The data supporting the findings of this study are available from the corresponding author upon reasonable request.

## Abbreviations

The following abbreviations are used in this manuscript:

ANCOVA	Analysis of Covariance
CIs	Confidence Intervals
COREQ	The Qualitative Research Reporting Consolidated Criteria
MAAS	Mindful Attention Awareness Scale
MD	Mean Difference
NIC	Nursing Interventions Classification
Ö	Student’s code
PSS	Perceived Stress Scale
TIDieR	Template for Intervention Description and Replication

## Appendix A

**Table A1:** Research Content and Yoga Sequence.

Weeks	Topics
Week 1	The history and philosophy of yoga (90 min.)
Week 2	The effects of yoga on health and evidence-based studies (90 min.)
Week 3–14	Yoga practice (90 min.)
Yoga Sequence (Asanas/Poses)	
- Chakravakasana (Cat-Cow Poses) - Tadasana (Mountain Pose) - Urdhva Hastasana (Raised Hands Pose) - Uttanasana (Standing Forward Bend) - Ardha Uttanasana (Half Forward Bend) - Plank - Ashtang Pranam - Bhujangasana (Mini Cobra) - Adho Mukha Svanasana (Downward Dog) - Vrksasana (Tree Pose) - Virabhadrasana I (Warrior I) - Virabhadrasana II (Warrior II) - Utkatasana (Chair Pose) - Salabhasana - Paschimottanasana (Seated Forward Bend) - Dandasana - Viparita Virabhadrasana (Reverse Warrior) - Setubandhasana (Bridge Pose) - Child’s pose - Ananda Balasana (Happy Baby Pose) - Windshield Wipers Pose - Savasana (Corpse Pose)	
